# Correlation of White Matter Hyperintensities and Perivascular Spaces With Montreal Cognitive Assessment (MoCA) Scores in Patients Evaluated for Anti-amyloid Therapy

**DOI:** 10.7759/cureus.85832

**Published:** 2025-06-12

**Authors:** Saurabh Rohatgi, Jeremy N Ford, Shenghua Zhu, Benjamin M Kozak, Maryam Vejdani Jahromi, Harry R Griffin, Odette Ganem Chagui, Amol Dua, Esteban Calle Cadavid, Hana Farzaneh, Liliana Ramírez Gómez, Javier Romero

**Affiliations:** 1 Division of Neuroradiology, Department of Radiology, Massachusetts General Hospital, Harvard Medical School, Boston, USA; 2 Department of Neurology, Massachusetts General Hospital, Harvard Medical School, Boston, USA

**Keywords:** cognitive impairment, dementia, moca, perivascular spaces, white matter hyperintensities

## Abstract

Objective

This study aimed to investigate the relationship between white matter hyperintensities (WMH) and perivascular spaces (PVS) on magnetic resonance imaging (MRI) with Montreal Cognitive Assessment (MoCA) scores in patients referred for possible lecanemab therapy based on clinical suspicion of Alzheimer’s disease (AD) prior to biomarker confirmation.

Materials and methods

In this retrospective review, 149 consecutive patients with suspected AD between November 2023 and June 2024 who were evaluated for possible lecanemab therapy were identified. All underwent brain MRI and had valid MoCA scores. WMH were graded using the Fazekas scale (0-3). PVS were visually graded (1-4) in the basal ganglia and centrum semiovale on T2-weighted images. Generalized linear models assessed the association between imaging markers and MoCA, adjusting for age, sex, hypertension (HTN), hyperlipidemia (HLD), and diabetes mellitus (DM).

Results

The mean MoCA score was 19.56, reflecting mild to moderate cognitive impairment. The mean Fazekas score was 1.37, indicating mild to moderate WMH burden, while the mean PVS scores for basal ganglia and centrum semiovale were 1.99 and 2.37, respectively. There is no significant correlation between age and MoCA scores in our patient population. A negative association of -0.1 between the Fazekas score and MoCA score was observed after controlling for the effects of PVS. In contrast, PVS did not significantly correlate with MoCA score.

Conclusion

In patients evaluated for possible lecanemab therapy, a higher WMH burden was negatively associated with global cognition, whereas PVS demonstrated no significant relationship with MoCA scores. These findings suggest that WMH may be an imaging marker of vascular pathology in those suspected of AD.

## Introduction

Alzheimer’s disease (AD) represents a major cause of dementia worldwide, characterized by progressive cognitive decline and the accumulation of amyloid-beta (Aβ) plaques in the brain, manifesting initially as mild cognitive impairment (MCI) or mild dementia [1]. It affects around 50 million individuals worldwide, with the number projected to triple by 2050 [2].

Recent advances in anti-amyloid monoclonal antibodies, such as lecanemab, have been shown to reduce brain fibrillar amyloid and slow clinical decline in early-stage AD [3,4]. While individuals must meet the diagnostic criteria for MCI or mild dementia due to AD based on biomarker evidence of amyloid accumulation, either with amyloid positron emission tomography (PET) or cerebrospinal fluid (CSF) biomarkers, many patients are referred for possible AD and potential lecanemab therapy based on clinical suspicion [5].

Neuroimaging plays a pivotal role in the evaluation of suspected AD by characterizing vascular or structural pathologies that can exacerbate or mimic neurodegeneration. In conjunction with neuroimaging, global cognition is screened with the Montreal Cognitive Assessment (MoCA) [6] on a 30-point scale rather than the traditional Mini‑Mental State Examination (MMSE). Multiple head-to-head studies have demonstrated that MoCA is more sensitive to the subtle executive, visuospatial, and memory deficits that characterize prodromal and early-stage AD [7-10]. Accordingly, MoCA was selected as our primary cognitive outcome measure.

White matter hyperintensities (WMH) serve as a recognized radiologic marker of small vessel disease (SVD) and have been associated with disrupted cognitive function [11,12]. The severity of WMH burden has shown varying degrees of correlation with cognitive decline, particularly in executive function and processing speed [8,13].

Perivascular spaces (PVS), also referred to as Virchow-Robin spaces, are fluid-filled channels along penetrating vessels and can become visibly dilated on T2-weighted MRI. Although some studies have indicated a relationship between PVS burden and cognitive impairment, findings remain inconsistent [14-16]. PVS may also influence Aβ clearance and inflammatory responses, although the precise nature of this interaction is not fully established [17,18]. PVS could have practical implications for understanding cognitive decline in those suspected of AD.

Despite extensive literature on the roles of WMH and PVS in cognitive impairment, limited evidence exists regarding these markers in patients specifically referred for possible lecanemab therapy before confirmatory amyloid biomarker testing. Thus, the primary objectives of this study were (1) to evaluate the relationship between WMH and global cognition (assessed by the MoCA) and (2) to assess whether PVS in the basal ganglia versus centrum semiovale independently correlates with MoCA scores in a cohort of patients suspected of AD.

## Materials and methods

Study design and population

This retrospective study was approved by the Mass General Brigham Institutional Review Board (IRB) with a waiver of informed consent (approval number: 2024P000596). We identified 149 consecutive patients (73 males, 76 females) who were referred to our memory clinic between November 2023 and June 2024 for possible lecanemab therapy based on clinical suspicion of AD. Inclusion criteria required a documented MoCA score and a brain MRI within 12 months of clinical evaluation; no patient had amyloid-PET or CSF confirmation at the time of imaging.

Relevant demographic and clinical data, including vascular risk factors such as hypertension (HTN), hyperlipidemia (HLD), and diabetes mellitus (DM), were obtained from electronic medical records. The medical records were de-identified for the images, and the images were fully de-identified before analysis.

Cognitive assessment

Global cognition was assessed using the 30-point MoCA, which evaluates attention, memory, executive function, language, visuospatial ability, and orientation [6]. Permission for using MoCA was obtained from MoCA Test Inc., Québec, Canada.

MRI protocol

MRI scans were performed using both 1.5T and 3T scanners from GE (GE HealthCare, Chicago, IL) and Siemens (Siemens Healthineers, Erlangen, Germany). A typical MR protocol included the following sequences: axial T2, fluid-attenuated inversion recovery (FLAIR), T2*-weighted gradient-recalled echo (GRE), diffusion-weighted imaging (DWI), and T1-weighted anatomic imaging, with or without multi-echo susceptibility-weighted imaging (SWI). Slice thickness and imaging parameters varied slightly by scanner and hardware.

Image analysis

**Figure 1:**
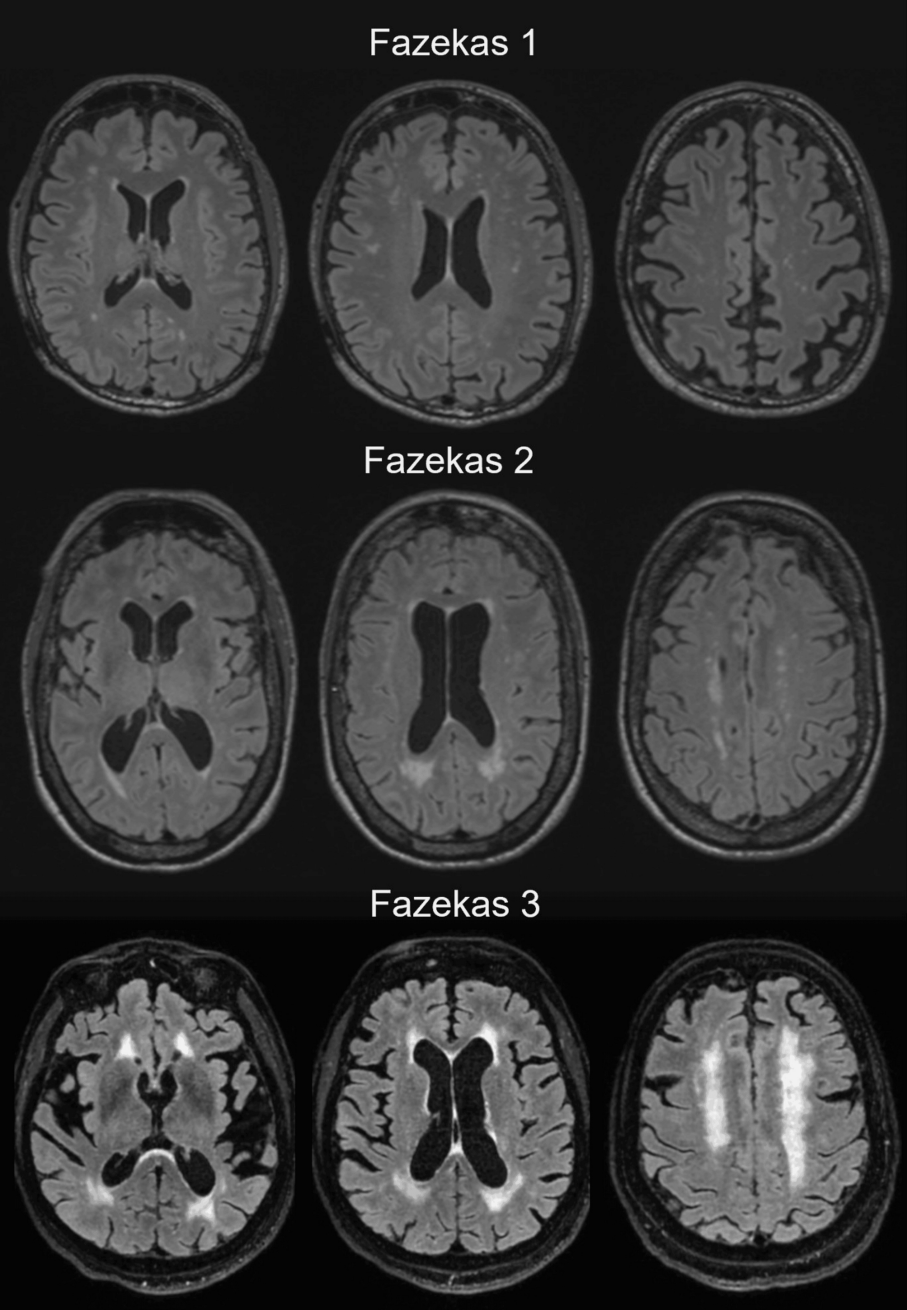
Axial fluid-attenuated inversion recovery images (FLAIR) illustrating the Fazekas score.

Six board-certified radiologists, blinded to the specific study hypothesis, evaluated the MRI images using Visage 7, version 7.1.9c (Visage Imaging Inc., San Diego, CA). Lacunar infarcts were excluded from the following assessments. WMHs were graded using the Fazekas scale (0-3) per the Standards for Reporting Vascular Changes (STRIVE) [19]. Grade 0 represented minimal or no WMH, Grade 1 indicated mild punctate WMH, Grade 2 indicated moderate lesions forming bridging or confluent areas, and Grade 3 indicated large confluent lesions (Figure 1).

**Figure 2:**
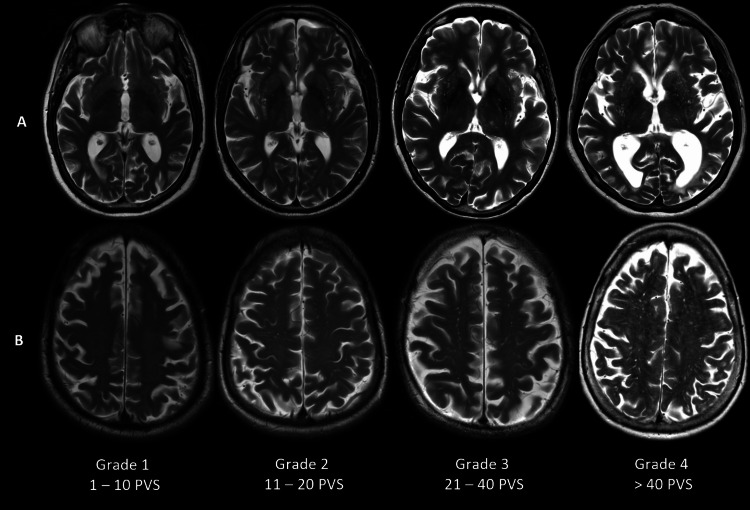
Axial T2-weighted images showing dilated perivascular spaces (PVS) in the basal ganglia (a) and centrum semiovale (b) from Grade 1 to Grade 4.

PVS were assessed using a four-point visual scale (1-4) in the basal ganglia and centrum semiovale described by Potter et al. using T2-weighted images [14]. Higher grades indicated a greater number of dilated spaces. PVS were distinguished from WMH based on T2 and FLAIR signal characteristics, with PVS being isointense to CSF on T2 and showing minimal or no signal on FLAIR. Grade 1 corresponded to 1-10 dilated PVS; Grade 2 to 11-20; Grade 3 to 21-40; and Grade 4 to >40 dilated PVS (Figure 2). Despite the majority of examinations being performed at 3T, there was no significant difference in visual PVS scores between 1.5 Tesla (T) and 3 T.

In cases of disagreement, lesions were reassessed by consensus with a third evaluator.

Statistical analysis

All our statistical evaluations were conducted in the Spyder environment (https://www.spyder-ide.org), compatible with Python 3.10 (Python Software Foundation (PSF), Beaverton, OR). Demographic and imaging variables were summarized using mean and standard deviation (SD) at a 95% confidence interval (CI) or percentages. The frequency and distribution of MoCA scores were analyzed across the patient population to assess cognitive impairment. Pearson’s correlation was used to evaluate the relationship between age and MoCA scores.

To quantify the impact of cardiovascular risk factors, including HTN, DM, and HLD, on the dependent variables, independent t-tests between each risk factor and the Fazekas score and the MoCA score were used.

To assess the independent relationship between WMH and MoCA scores, a generalized linear model regression was performed while controlling for the confounding effects of age, sex, HTN, HLD, DM, PVS-basal ganglia, and PVS-centrum semiovale. Similar generalized linear models were used, and MoCA was regressed on (i) PVS-basal ganglia and (ii) PVS-centrum semiovale, controlling for the effects of various confounders, including age, sex, HTN, HLD, DM, and WMH. A significant threshold of p < 0.05 was set for all statistical evaluations.

## Results

A total of 149 patients (73 males, 76 females; mean age 72.6 ± 7.7 years) were included. One hundred and fifteen patients were actually diagnosed with AD based on either amyloid PET and/or CSF analyses. Sixteen patients had a clinical diagnosis of AD without any confirmatory tests done. Among those 131 patients with AD, 61 patients were eligible for lecanemab treatment, of which 52 received infusion.

The mean MoCA score was 19.6 ± 5.1, spanning mild to moderate impairment as shown in Table 1.

**Table 1:** Demographic characteristics of the study group MocA: Montreal Cognitive Assessment; PVS: perivascular space

Characteristics	Value (SD)	95% CI
Total patients included	149	N/A
Female	76 (51.8%)	N/A
Age (in years)		
Mean	72.6 (7.7)	71.4-73.9
Median	74	72-75
Range	50-89	N/A
Baseline cognition		
MoCA	19.6 (5.1)	18.7-20.4
Comorbidities		
Hypertension	92 (61.7%)	N/A
Diabetes	18 (12.1%)	N/A
Dyslipidemia	125 (83.9%)	N/A
Fazekas	1.38 (0.77)	1.25-1.5
PVS basal ganglia	1.99 (0.82)	1.86-2.13
PVS centrum semiovale	2.37 (0.95)	2.22-2.52

**Figure 3:**
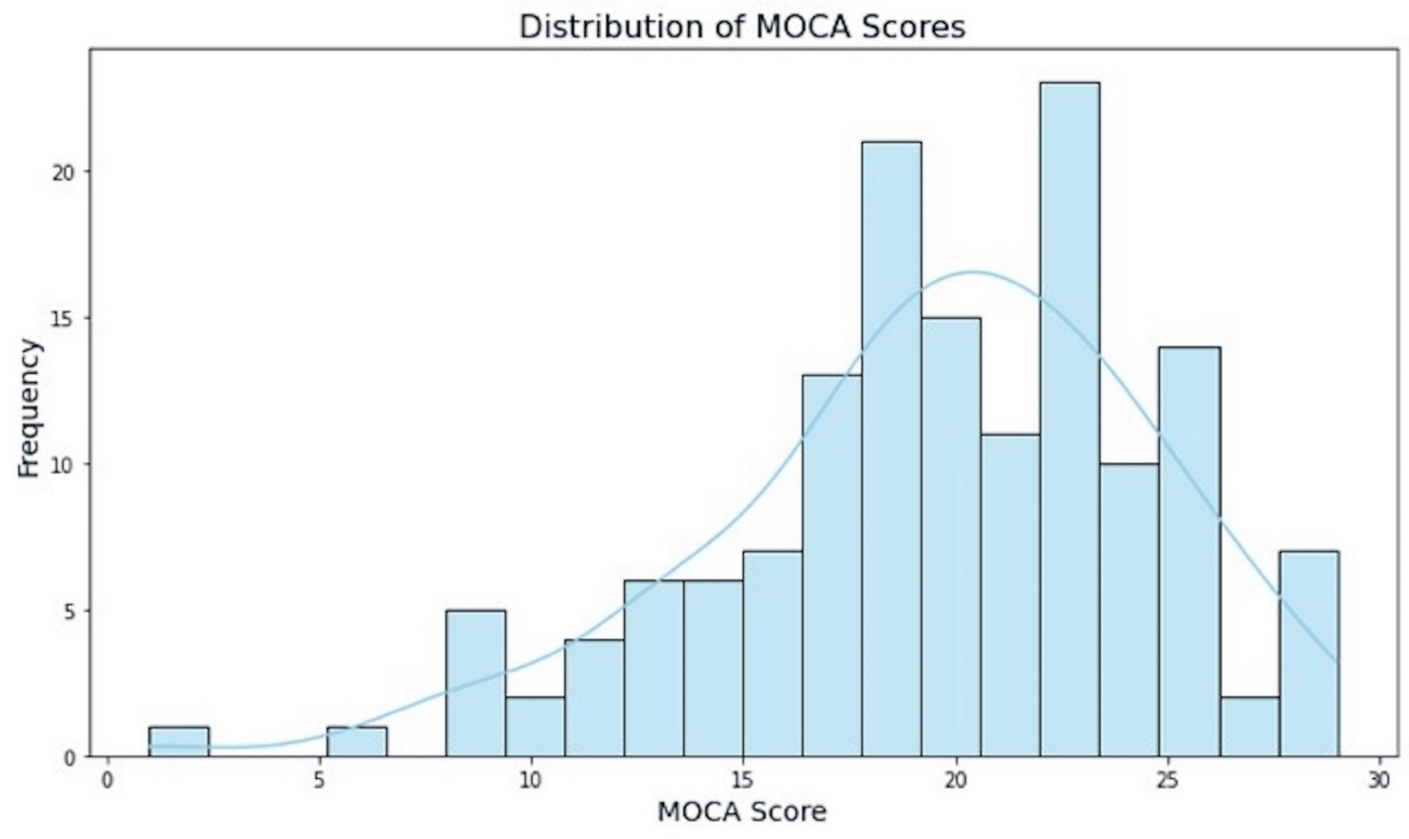
Frequency and distribution of Montreal Cognitive Assessment (MoCA) scores

The frequency and distribution of the MoCA score are summarized in Figure 3.

**Figure 4:**
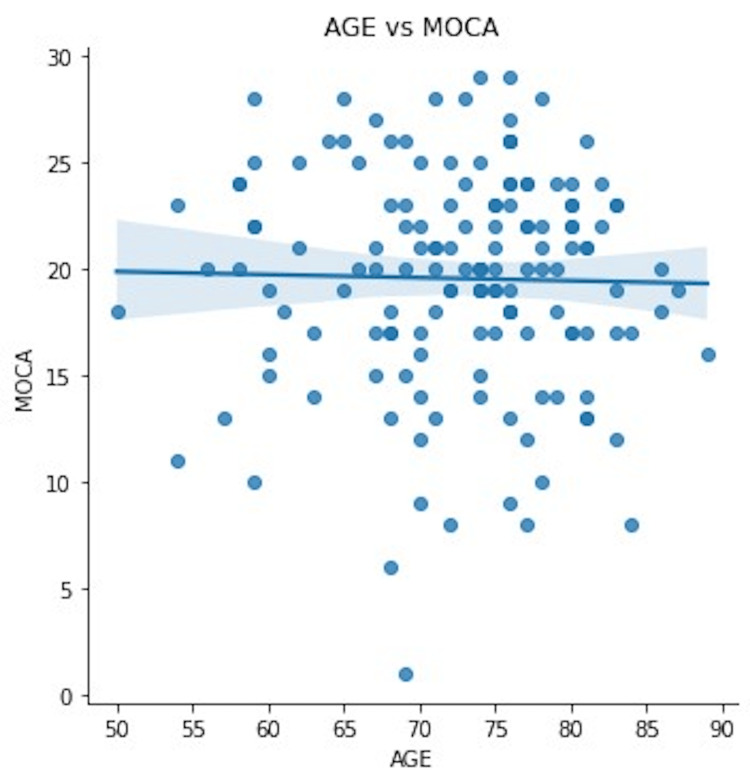
Correlation analysis of age and Montreal Cognitive Assessment (MoCA) score

No significant correlation emerged between age and MoCA score (r = -0.0215, p = 0.79) (Figure 4).

The mean Fazekas score was 1.38 ± 0.77, indicating a mild to moderate WMH burden. PVS were graded separately in the basal ganglia and centrum semiovale, yielding mean scores of 1.99 ± 0.82 and 2.37 ± 0.95, respectively (Table 1). A comprehensive characterization of the distribution of Fazekas scores and PVS grades on baseline imaging is summarized in Table 2.

**Table 2:** Distribution of white matter hyperintensities Fazekas scores and perivascular space (PVS) grades in basal ganglia and centrum semiovale on baseline imaging.

Fazekas and PVS grades	N (%)
Fazekas	
Grade 0	9 (6.0%)
Grade 1	92 (61.7%)
Grade 2	31 (20.8%)
Grade 3	17 (11.4%)
PVS basal ganglia	
Grade 1	44 (29.5%)
Grade 2	68 (45.6%)
Grade 3	31 (20.8%)
Grade 4	6 (4.0%)
PVS centrum semiovale	
Grade 1	27 (18.1%)
Grade 2	62 (41.6%)
Grade 3	38 (25.5%)
Grade 4	22 (14.8%)

**Figure 5:**
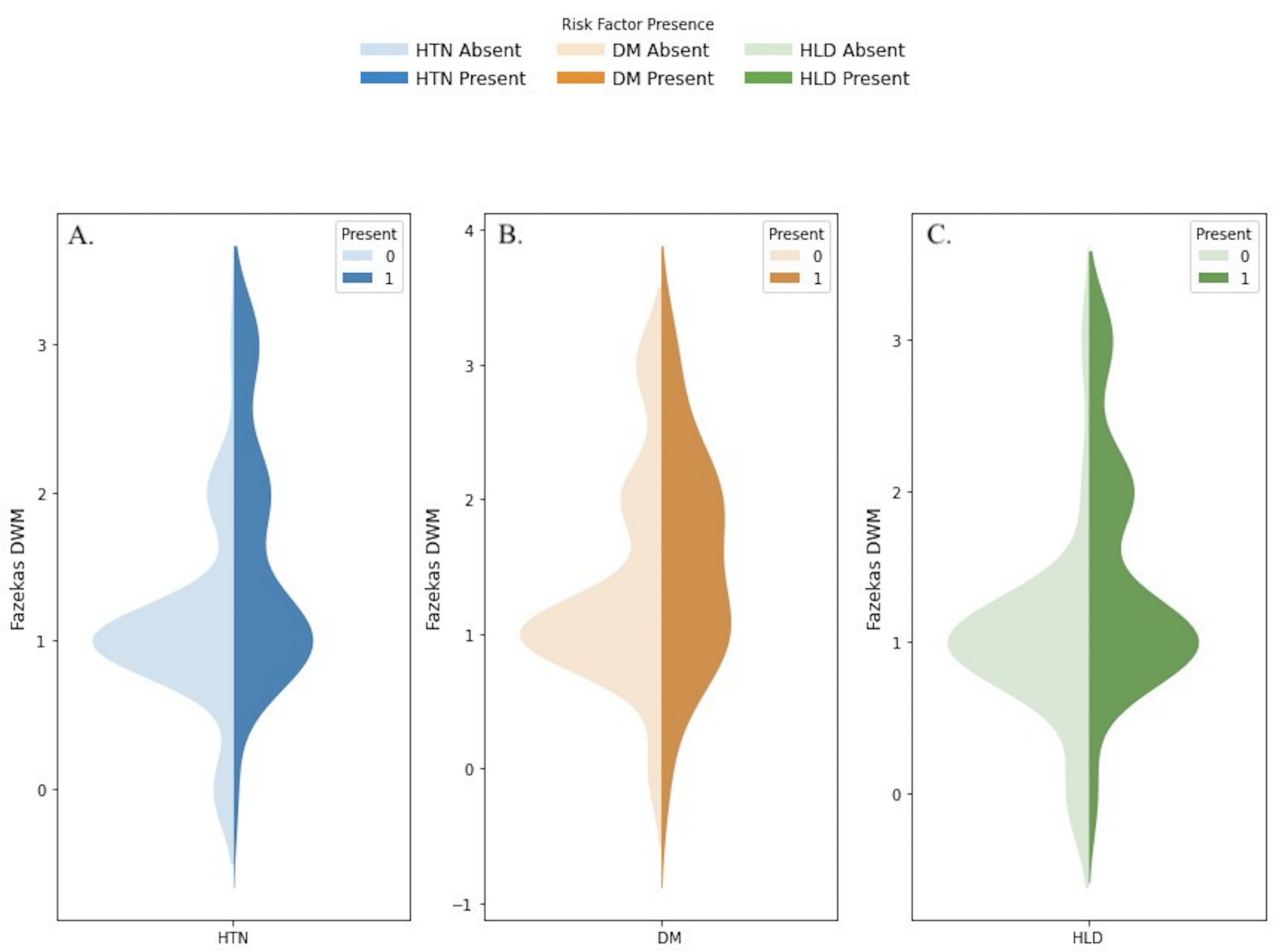
Violin plot depicting the Fazekas distribution with various cardiovascular risk factors A. Fazekas distribution with hypertension (HTN): Light blue represents people without HTN, while dark blue represents people with it. B. Fazekas distribution with diabetes mellitus (DM): Light orange represents people without DM, while dark orange represents those with it. C. Fazekas distribution with hyperlipidemia (HLD): Light green represents people without HLD, while dark green represents those with it. DWM: deep white matter

A large proportion of the cohort had HTN (61.7%) and HLD (83.9%), while a smaller percentage had DM (12.1%) (Table 1). The independent t-tests showed that hypertension and hyperlipidemia were significantly associated with higher Fazekas scores (Table 3). However, diabetes mellitus was not significantly associated with Fazekas scores (Table 3). A violin plot depicting the Fazekas distribution with various cardiovascular risk factors is summarized in Figure 5.

**Table 3:** Distribution of cardiovascular risk factors and association with Fazekas and Montreal Cognitive Assessment (MoCA) scores

Cardiovascular risk factor	Percentage population with risk factor	T-test with Fazekas		T-test with MoCA	
		T value	P value	T value	P value
Hypertension (HTN)	61.7%	4.02	<0.001	1.48	0.13
Diabetes mellitus (DM)	12.1%	1.06	0.3	0.78	0.43
Hyperlipidemia (HLD)	83.9%	2.67	<0.01	0.67	0.5

None of the cardiovascular risk factors had a significant relationship with MoCA scores, implying that while these conditions influence brain structure, they may not directly impact cognitive performance.

**Figure 6:**
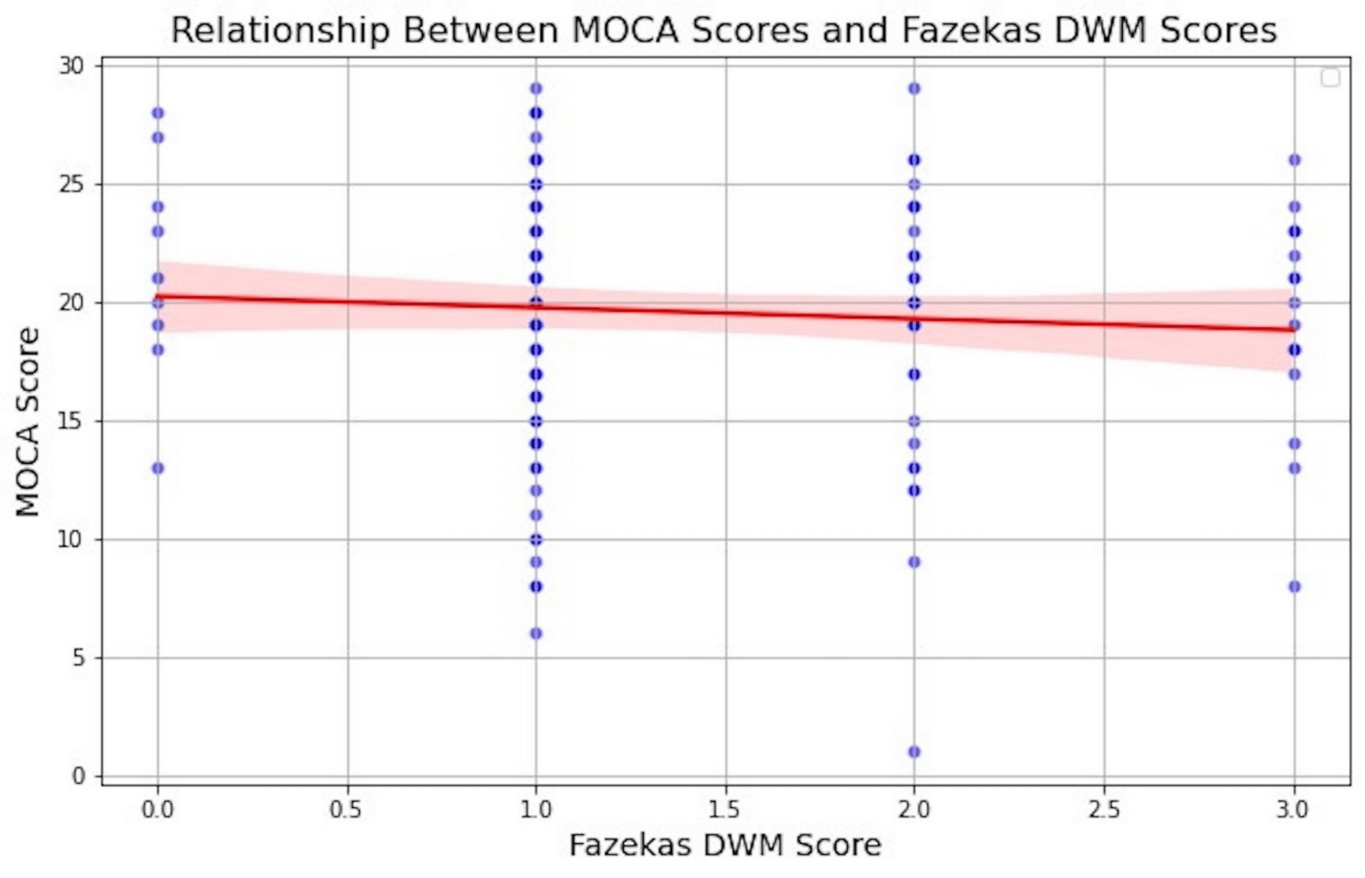
Generalized linear model regression between Fazekas deep white matter (DWM) and Montreal Cognitive Assessment (MoCA), while controlling for the confounding effects of age, sex, hypertension, hyperlipidemia, diabetes mellitus, perivascular space (PVS)-basal ganglia, and PVS-centrum semiovale. This graph shows a negative association of -0.1 between the Fazekas DWM score and MoCA score (p-value: 0.019).

A regression model adjusting for age, sex, HTN, HLD, DM, and PVS indicated a modest yet statistically significant inverse relationship between the Fazekas score and MoCA score (β = -0.1, p = 0.019) (Figure 6).

In contrast, neither PVS in the basal ganglia nor the centrum semiovale showed a significant association with MoCA score once the WMH burden was taken into account (p = 0.18 and p = 0.27, respectively).

## Discussion

As the population ages, numerous changes in the brain parenchyma have a negative impact on cognition. Our goal was to identify imaging biomarkers for cognitive impairment in patients undergoing evaluation for lecanemab eligibility. In this cohort of older adults considered for possible lecanemab therapy based on clinical suspicion of AD, we observed a modest negative correlation between WMH burden and global cognition (MoCA score). Although the effect size was relatively small, it reached statistical significance and implicates small vessel disease in cognitive impairment [8,13]. Because the majority of patients with AD exhibit cerebral amyloid angiopathy (CAA), these WMH likely reflect CAA-related microangiopathy rather than hypertensive arteriolosclerosis [20]. Importantly, WMHs are not the sole vascular lesion influencing cognition. For example, Warren and colleagues, analyzing data from the Dallas Heart Study, reported that the number of lacunar infarcts, rather than total WMH volume, showed the strongest association with lower cognitive scores [21]. Likewise, Birdsill et al. demonstrated in 349 community-dwelling adults that WMH localized to the superior corona radiata correlated with slower cognitive speed and reduced flexibility after age adjustment, whereas WMH did not relate to memory performance [22]. Together, these studies highlight that the cognitive impact of small‑vessel disease is lesion-specific and tract-dependent. Our global Fazekas score may capture only part of this complex relationship.

Conversely, PVS severity was not independently related to MoCA scores after adjustment for WMH, implying that simple PVS counts add little prognostic value for cognitive performance in this clinical context. The broader literature on PVS and cognition is notably inconsistent. Serra et al. found no association between PVS load and either global cognition or the risk of dementia/AD [23], whereas Libecap et al. reported a modest inverse relationship between PVS burden and MoCA scores in community-dwelling older adults [24]. A large meta-analysis of 8,395 individuals by Francis et al. further complicated the picture, showing that PVS volume correlates strongly with advancing age but less consistently with cognition once age is taken into account [25]. Several factors could explain these observed discrepancies. First, the heterogeneity of MRI field strengths (1.5T vs. 3T) may have obscured subtle differences in PVS. Second, the MoCA scores in our cohort spanned a range that included both mild MCI-like features and more pronounced deficits, potentially overshadowing the contribution of PVS. Third, many patients likely had underlying AD-type pathology, but without confirmatory PET or CSF data, we could not precisely stratify them by amyloid or tau burden.

A large proportion of our cohort had HTN and HLD, and control of these cardiovascular risk factors continues to be important in the prevention of SVD, even in patients who are eligible for anti-amyloid treatments.

Our results highlight the potential utility of WMH as an imaging marker of vascular pathology in patients suspected of AD. Even without definitive amyloid biomarkers, WMH might reflect comorbid vascular changes that exacerbate or mimic AD-related cognitive decline. Clinically, the findings suggest that WMH grading could enrich risk stratification and may be more vulnerable to subsequent cognitive decline.

We must note some limitations of our study, including inherent biases given the retrospective nature and referral pattern of a memory clinic, which introduces selection bias and limits generalizability. Our sample size did not account for confounding comorbidities such as body mass index, genetic factors such as apolipoprotein E (APOE) genotype, and whole brain and/or regional atrophy, as well as the presence of infarcts. MRI parameters varied by scanner type, field strength, and slice thickness, which likely added measurement noise for PVS assessment. We relied on visual rating scales rather than automated volumetric methods, potentially overlooking subtle regional effects or partial-volume WMH. Additional automated volumetric studies may increase sensitivity in assessing more robust associations and provide better inter-rater agreement. Future work on longitudinal studies incorporating volumetric WMH, automated PVS metrics, and regional atrophy maps will clarify how combined vascular and neurodegenerative burdens shape cognitive trajectories and treatment response. In the time to come with machine learning frameworks, we can better assess data time points based on clinical history, symptomatology, and imaging using more robust screening tools that can parse out the etiologies of cognitive decline.

## Conclusions

Among patients suspected of AD and evaluated for lecanemab therapy, higher WMH (as measured by the Fazekas scale) demonstrated a modest but statistically significant inverse correlation with cognitive impairment. In contrast, PVS burden in either the basal ganglia or the centrum semiovale was not independently associated with cognitive performance. Routine WMH assessment may therefore aid risk stratification in real‑world therapeutic decision‑making. Future prospective studies incorporating amyloid and tau biomarkers, volumetric imaging measures, and region-specific WMH analyses are needed to fully clarify the role of vascular pathology in AD and to inform targeted therapeutic strategies.
